# ALMT12 interacts with and inhibits SLAC1 to modulate stomatal movements and enhance plant biomass

**DOI:** 10.1093/plphys/kiaf460

**Published:** 2025-09-29

**Authors:** Ping Lin, Hui Zhou, Qing Zhao, Liumei Li, Jiamei Liu, Zhuoran Hu, Yunxin Luo, Cuizhu Feng, Yu Long

**Affiliations:** State Key Laboratory of Crop Stress Adaptation and Improvement, School of Life Sciences, Henan University, Kaifeng 475001, China; State Key Laboratory of Crop Stress Adaptation and Improvement, School of Life Sciences, Henan University, Kaifeng 475001, China; State Key Laboratory of Crop Stress Adaptation and Improvement, School of Life Sciences, Henan University, Kaifeng 475001, China; State Key Laboratory of Crop Stress Adaptation and Improvement, School of Life Sciences, Henan University, Kaifeng 475001, China; State Key Laboratory of Crop Stress Adaptation and Improvement, School of Life Sciences, Henan University, Kaifeng 475001, China; State Key Laboratory of Crop Stress Adaptation and Improvement, School of Life Sciences, Henan University, Kaifeng 475001, China; State Key Laboratory of Crop Stress Adaptation and Improvement, School of Life Sciences, Henan University, Kaifeng 475001, China; State Key Laboratory of Crop Stress Adaptation and Improvement, School of Life Sciences, Henan University, Kaifeng 475001, China; State Key Laboratory of Crop Stress Adaptation and Improvement, School of Life Sciences, Henan University, Kaifeng 475001, China

## Abstract

Stomata are pores that control carbon dioxide (CO_2_) and water exchange by modulating their aperture in response to different environmental and internal signals. The Slow-type SLOW ANION CHANNEL-ASSOCIATED 1 (SLAC1) and Rapid-type ALUMINUM-ACTIVATED MALATE TRANSPORTER 12 (ALMT12) anion channels mediate anion efflux in guard cells to promote stomatal closure. These channels were previously thought to function as 2 independent anion-permeable pores differing in their activation kinetics, voltage dependence, and anion selectivity. In this study, we found that ALMT12 interacts with and represses SLAC1 anion permeability in *Xenopus* oocytes and Arabidopsis (*Arabidopsis thaliana*) guard cells. This channel–channel regulatory mechanism modulates stomatal movements under high CO_2_ conditions and enhances plant biomass.

## Teaser

ALMT12 interacts with SLAC1 and modulates stomatal movements with 2 roles, promoting stomatal closure as R-type anion channel while suppressing it as SLAC1 inhibitor.

## Introduction

The continuous consumption of fossil fuels over the past century has raised atmospheric CO_2_ levels and leading to frequent occurrences of extreme climate events. These changing environmental conditions impose constraints on modern crops due to their lack of adaptation (Cao et al. 2010). Stomata are the pores mediating both CO_2_ uptake and water transpiration, serving as a key component of the Soil–Plant–Atmosphere Continuum, playing a key role in both increasing plant biomass and reducing water transpiration (Blatt et al. 2017; Endo and Torii 2019). As such, stomata are critical for plant adaptation to environmental changes (Buckley 2005; Martínez-Vilalta and Garcia-Forner 2017). They have accompanied plants since their colonization of land, enduring numerous drastic shifts in global climate (Chen et al. 2017; Clark et al. 2022).

Stomata regulate gas exchange and water transpiration by their opening and closing in response to environmental changes (Wang et al. 2022). These behaviors are controlled by changes in turgor pressure dictated by ion fluxes across the guard cell membrane (Kim et al. 2010; Wang et al. 2014). In particular, 2 types of anion channels play important roles in stomatal closure by mediating anion efflux (Li et al. 2024). In 2008, the Slow-type (S-type) SLOW ANION CHANNEL-ASSOCIATED 1 (SLAC1) was identified through a mutant screen for CO_2_ insensitive stomata (Negi et al. 2008). Later, ALUMINUM-ACTIVATED MALATE TRANSPORTER 12 (ALMT12), also called QUICK-ACTIVATING ANION CHANNEL 1 (QUAC1), was identified as the Rapid-type (R-type) anion channel (Meyer et al. 2010; Sasaki et al. 2010; Merilo et al. 2013; Hõrak et al. 2016; Yamamoto et al. 2016). Although both channels promote stomatal closure upon activation via phosphorylation in response to the stress phytohormone abscisic acid (ABA), hydrogen peroxide (H_2_O_2_), and calcium ions (Ca^2+^) (Kim et al. 2010; Hedrich 2012; Imes et al. 2013; Qin et al. 2022), they function as distinct anion-permeable pores with differential characteristics beyond their activation speed. On one hand, SLAC1 forms a trimeric channel mediating Cl^−^ and NO_3_^−^ efflux (Li et al. 2022), whereas ALMT12 functions as a dimeric channel mediating malate efflux (Qin et al. 2022). On the other hand, SLAC1 plays a more important role in stomatal closure than ALMT12, as *slac1* loss-of-function mutants exhibits a more severe impairment in stomatal closure than the *almt12* mutant (Hsu et al. 2021; Jalakas et al. 2021) However, the relationship between these 2 type channels remains unclear.

Here, we demonstrated that the 2 channels, SLAC1 and ALMT12, physically interact to inhibit SLAC1 channel activity. Furthermore, this channel–channel regulation results in distinct ion fluxes in response to environmental signals, which highlights their importance in the regulation of stomatal movements.

## Results

### ALMT12 inhibits SLAC1 activity

Previous studies have reported that SLAC1 interacts with the POTASSIUM (K^+^) CHANNEL IN ARABIDOPSIS THALIANA 1 (KAT1) and inhibits its conductance (Zhang et al. 2016), suggesting channel–channel interaction as a potential mode for SLAC1 in guard cells. We hypothesized that the 2 types of anion channels in guard cells might interact and participate in reciprocal regulation.

**Figure 1.**
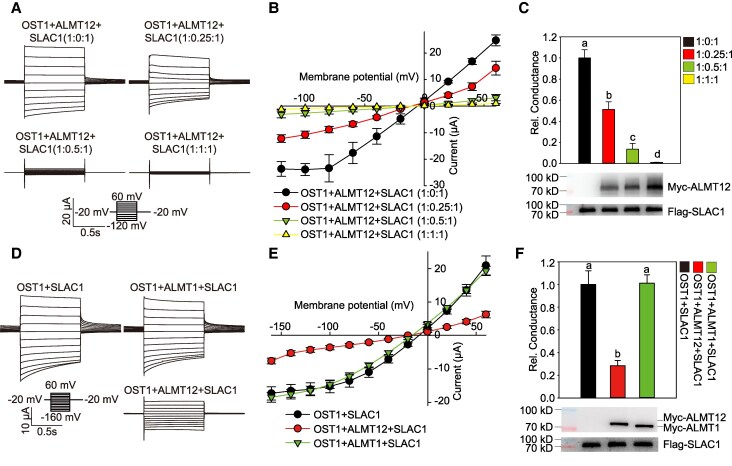
ALMT12 inhibits the anion channel activity of SLAC1. **A** to **C)** TEVC recording in *X.* oocytes. Whole-cell current recording **A)**, steady-state current-voltage relationships **B)**, and relative conductance and immunoblotting analysis **C)** in oocytes co-injected with gradient amount of *ALMT12* cRNA (0, 1.05, 2.09, 4.17 and 8.83 ng) with the same amount of *OST1* and *SLAC1* cRNA (8.33 ng), as the ratio indicated in **A)** to **C)**; conductance of *OST1*  *+*  *SLAC1* was set to 1; the data are means ± Se (*n* ≥ 11). **D)** Whole-cell currents recorded in oocytes co-expressing *SLAC1*, *OST1*, and *ALMT12* (or *ALMT1*). **E** and **F)** Steady-state current-voltage relationships **E)** and relative conductance and immunoblotting analysis **F)** of oocytes recorded in **D)**. Conductance of *OST1* + *SLAC1* was set to 1; the data are means ± Se (*n* ≥ 11). Voltage protocols and the time and current scale bars for the recordings are shown in **A)** and **D)**. Bars with different letters indicate significant differences at *P* < 0.05 by ANOVA with Tukey's comparison test.

SLAC1 exhibits channel activity when its complementary RNA (cRNA) is co-expressed with that of *OPEN STOMATAL 1* (*OST1*) in *Xenopus laevis* oocytes (Geiger et al. 2009), as we show here using a 2-electrode voltage clamp (TEVC). Interestingly, SLAC1 activity was inhibited by co-injection of *ALMT12* cRNA (Fig. 1, A to C). Additionally, the degree of SLAC1 inhibition increased with higher quantities of injected *ALMT12* cRNAs (Fig. 1, A to C). These results suggest that ALMT12 inhibits SLAC1 channel activity.

Further, we examined the specificity of ALMT12-mediated suppression of SLAC1 activity by co-injecting the cRNA of *ALUMINUM-ACTIVATED MALATE TRANSPORTER 1* (*ALMT1*, a homolog of *ALMT12*) and found that ALMT1 had no effect on SLAC1 activity (Fig. 1, D to F). Additionally, western blot analysis showed that neither ALMT12 nor ALMT1 affected SLAC1 protein expression (Fig. 1F). These results indicate that ALMT12 specifically inhibits SLAC1 activity.

### ALMT12 interacts with SLAC1

**Figure 2.**
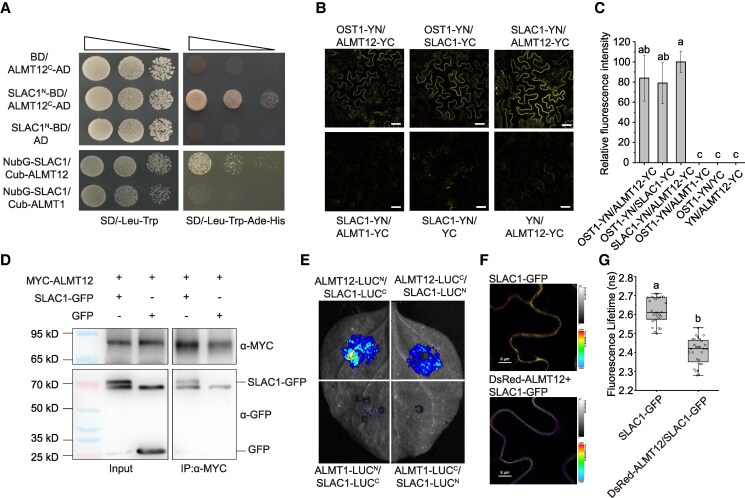
ALMT12 physically interacts with SLAC1. **A)** Interaction between the cytosolic domains of SLAC1 (SLAC1^N^) and ALMT12 (ALMT12^C^) examined by Y2H assay. Empty vector (BD and AD) was used as control. Interaction between the full-length SLAC1 (NubG-SLAC1) and the full-length ALMT12 (Cub-ALMT12) examined by membrane-based split-ubiquitin Y2H system. The full-length ALMT1 (Cub-ALMT1) was used as control. SD/-Leu-Trp, synthetic defined medium lacking Leu and Trp; SD/-Leu-Trp-Ade-His, SD medium lacking Leu, Trp, Ade and His. **B** and **C)** ALMT12 and SLAC1 interact in *N. benthamiana* leaves, as demonstrated by BiFC assays based on split-YFP. The combinations OST1-YN + ALMT12-YC and OST1-YN + SLAC1-YC were used as positive controls; ALMT1 and empty vectors were used as negative control. Scale bars, 50 *μ*m. The statistics for BiFC data were analyzed in **C)**. Data are means ± Se (*n* = 4–12). **D)** Interaction between SLAC1 and ALMT12 by Co-IP. GFP or GFP-tagged SLAC1 and MYC-tagged ALMT12 were transiently co-expressed in *N. benthamiana* leaves, and proteins were immunoprecipitated using anti-MYC antibody-conjugated agarose beads. The resulted precipitates were detected using anti-GFP and anti-MYC antibodies, respectively. IP, immunoprecipitation. **E)** Interaction between ALMT12 and SLAC1 in *N. benthamiana* leaves using LCI assays based on split-LUC; ALMT1 was used as negative control. These were performed independently at least 3 times. **F)** FLIM-FRET assay for the interaction between SLAC1-GFP and DsRed-ALMT12. **G)** The fluorescence lifetime of the SLAC1-GFP/DsRed-ALMT12 and SLAC1-GFP. The data are plotted with box and whiskers plots: whiskers plot represents minimum and maximum values, and box plot represents second quartile, median, and third quartile; *n* ≥ 27. Bars with different letters indicate significant differences at *P* < 0.05 by ANOVA with Tukey's comparison test.

We then examined the interaction between SLAC1 and ALMT12 using yeast 2-hybrid (Y2H) assays and membrane-based split-ubiquitin. The Y2H assay detected the interaction between the cytosolic domains of SLAC1 (SLAC1^N^) and ALMT12 (ALMT12^C^), while the membrane system detected the interaction between the full-length SLAC1 (NubG-SLAC1) and ALMT12 (Cub-ALMT12) (Fig. 2A). Nevertheless, no interaction between SLAC1 and ALMT1 was observed, which was consistent with the anion conductance activity assays (Fig. 1, D to F).

We then performed bimolecular fluorescence complementation (BiFC) assays. By co-expressing constructs encoding SLAC1 fused to the N-terminal half of yellow fluorescent protein (YFP; YN) and ALMT12 fused to the C-terminal half of YFP (YC) in *Nicotiana benthamiana* leaves. OST1, known to interact with both SLAC1 and ALMT12, was used as a positive control, and ALMT1 was used as a negative control. Consistent with previous studies (Geiger et al. 2009; Imes et al. 2013), OST1 interacted with both ALMT12 and SLAC1 (Fig. 2B). In addition, we observed reconstituted YFP fluorescence at the plasma membrane (PM) of epidermal cells upon co-expression of *SLAC1-YN* and *ALMT12-YC*, whereas no signal was detected when *SLAC1-YN* was paired with *ALMT1-YC* or an empty vector control (Fig. 2, B and C). These results are consistent with SLAC1 interaction with ALMT12.

The interaction between ALMT12 and SLAC1 was then verified by a co-immunoprecipitation (Co-IP) assay in which GFP or GFP-tagged SLAC1 and MYC-tagged ALMT12 were co-expressed in *N. benthamiana* leaves and immunoprecipitated with MYC agarose beads. We detected a SLAC1-GFP signal, while the GFP control showed no detectable signal (Fig. 2D).

In addition, we performed split-luciferase complementation imaging (LCI) assays in which ALMT12 and SLAC1 fused to the N- or C-terminal half of LUC (LUC^N^ or LUC^C^) were co-expressed in *N. benthamiana* leaves, and ALMT1 was used as negative control. Following the application of luciferin, a clear LUC signal was observed in leaf area co-expressing ALMT12 and SLAC1, but not in the negative control area (Fig. 2E).

Finally, fluorescence lifetime imaging microscopy-Förster resonance energy transfer (FLIM-FRET) assay was confirmed the interaction between the 2 channels. We observed FRET event between SLAC1-GFP and DsRed-ALMT12 at the PM (Fig. 2, F and G). Taken together, these results indicate that SLAC1 and ALMT12 interacted at the PM.

### ALMT12 inhibits SLAC1 with both transmembrane and cytosolic domain independently of OST1

**Figure 3.**
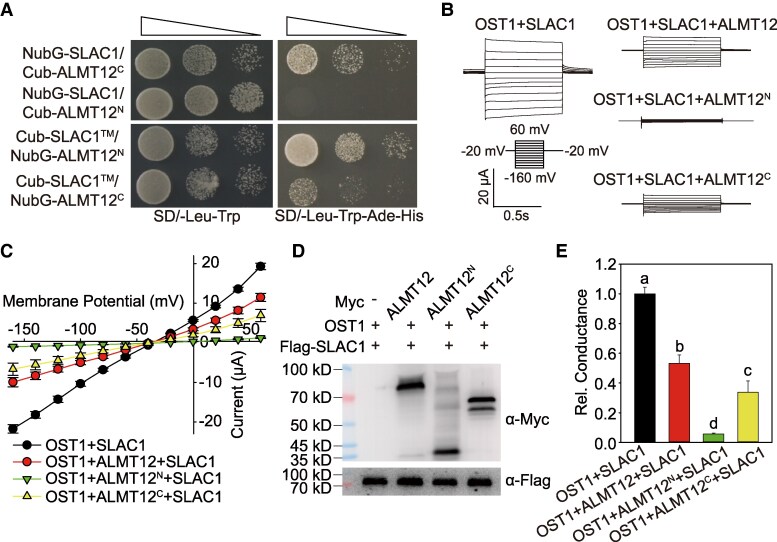
ALMT12 inhibits SLAC1 with both transmembrane and cytosolic domain. **A)** Membrane-based split-ubiquitin Y2H system showing the interaction between the full-length SLAC1 (NubG-SLAC1) and the cytosolic (Cub-ALMT12^C^) and transmembrane (Cub-ALMT12^N^) domains of ALMT12. The interaction between the transmembrane of SLAC1 (Cub-SLAC1^TM^) and the cytosolic (NubG-ALMT12^C^) and transmembrane (NubG-ALMT12^N^) domains of ALMT12. **B** to **E)** Whole-oocyte SLAC1 currents recording **B)**, steady-state current-voltage relationship **C)**, immunoblotting analysis **D)**, and relative conductance **E)** in oocytes co-injected with *SLAC1* and *OST1*, with *ALMT12*, *ALMT12^C^*, or *ALMT12^N^* cRNAs. The data in **C)** and **E)** are means ± Se (*n* ≥ 10). Bars with different letters indicate significant differences at *P* < 0.05 by ANOVA with Tukey's comparison test.

To characterize the inhibition between ALMT12 and SLAC1 in more details, we investigated the interactions between the cytosolic and transmembrane domains of ALMT12 and SLAC1 using the membrane-based split-ubiquitin Y2H system. The full-length SLAC1 channel interacted with the cytosolic domain (ALMT12^C^) of ALMT12, but not with its transmembrane domain (ALMT12^N^). By contrast, the transmembrane domain of SLAC1 (SLAC1^TM^) showed stronger interaction with the transmembrane domain than with the cytosolic domain of ALMT12 (Fig. 3A). These results suggest that ALMT12 and SLAC1 may perform a transmembrane–transmembrane and cytosolic–cytosolic interaction with each other.

Building on these findings, we split ALMT12 into its transmembrane (ALMT12^N^) and cytosolic (ALMT12^C^) domains and tested their effects on SLAC1 in oocytes. TEVC assays revealed that the truncated proteins, ALMT12^N^ and ALMT12^C^, inhibited SLAC1 to different degrees. ALMT12^C^ induced SLAC1 inhibition comparable to full-length ALMT12, whereas ALMT12^N^ completely abolished SLAC1 activity (Fig. 3, B to E).

Based on these results, we hypothesized that the transmembrane domain of ALMT12 might bind to SLAC1 to strongly inhibit its activity, while the cytosolic domain might interact with and further inhibit SLAC1. To test this hypothesis, we generated a series of truncated and chimeric proteins of ALMT12 and ALMT1 to assess their inhibitory effects. First, we found that the ALMT1^C^ did not inhibit the channel activity of SLAC1 (Supplementary Fig. S1A). Next, we fused the transmembrane and cytosolic domains of ALMT12 and ALMT1 to create 2 chimeric constructions: ALMT12^TM^-ALMT1^Cyt^ and ALMT1^TM^-ALMT12^Cyt^. Notably, all chimeric proteins exhibited inhibition to SLAC1 (Supplementary Fig. S1B), indicating that both transmembrane and cytoplasmic domains are important for the inhibition.

To exclude the possibility that ALMT12 might affect the protein stability or subcellular location of SLAC1, we used *N. Benthamiana* leaves to transiently express SLAC1-GFP, individually or collectively with MYC-ALMT12. Immunoblot analysis and confocal microscopy revealed that ALMT12 did not affect the stability or PM location of SLAC1 (Supplementary Fig. S2).

**Figure 4.**
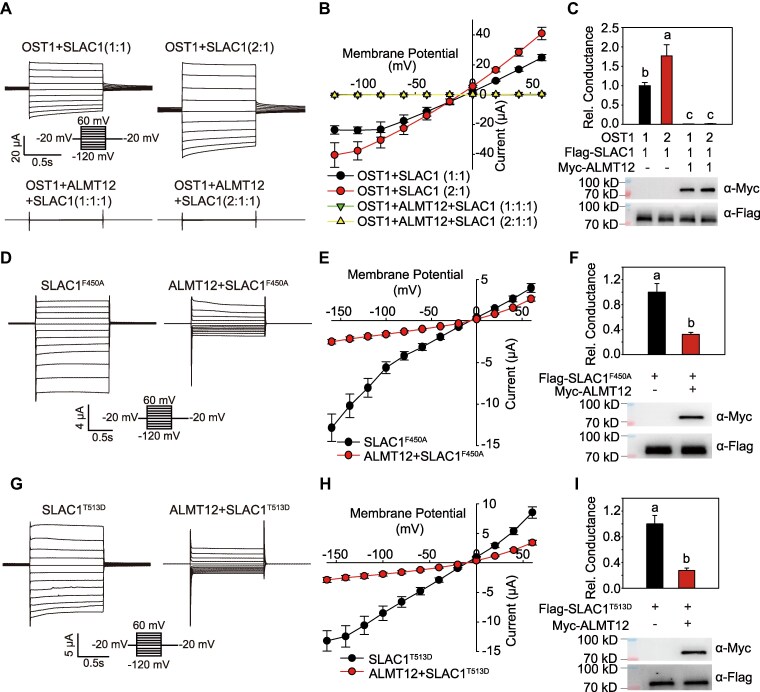
ALMT12 inhibits SLAC1 independently of OST1. **A** to **C)** Whole-cell current recording **A)**, steady-state current-voltage relationships **B)**, and relative conductance and immunoblotting analysis **C)** in oocytes co-injected with gradient amount of *OST1* cRNA (6.25 and 12.50 ng) with the same amount of *ALMT12* and *SLAC1* cRNA (6.25 ng), as the ratio indicated in **A)** to **C)**; the conductance of *OST1*  *+*  *SLAC1* set to 1. The data are means ± Se (*n* ≥ 9). **D** to **F)** Whole-cell current recording **D)**, steady-state current-voltage relationships **E)**, and relative conductance and immunoblotting analysis **F)** of oocytes expressing *SLAC1^F450A^* alone or with *ALMT12*; the conductance of *SLAC1^F450A^* set to 1. The data are means ± Se (*n* ≥ 12). **G** to **I)** Whole-cell current recording **G)**, steady-state current-voltage relationships **H)**, and relative conductance and immunoblotting analysis **I)** of oocytes expressing *SLAC1^T513D^* alone or with *ALMT12*; the conductance of *SLAC1^T513D^* set to 1. The data are means ± Se (*n* ≥ 13). Different letters indicate significant differences at *P* < 0.05 by ANOVA with Tukey's comparison test **C)** or Student's *t* test **F** and **I)**.

Both SLAC1 and ALMT12 are activated by OST1 (Geiger et al. 2009; Imes et al. 2013). Therefore, we investigated whether ALMT12 inhibited SLAC1 by competing for OST1 binding in oocytes. First, we injected the same and double amounts of *OST1* cRNA with *SLAC1* in oocytes, respectively. The TEVC assays showed that increasing *OST1* cRNA injecting amount enhanced the SLAC1 current but did not affect the inhibition (Fig. 4, A to C). Then, we tested 2 SLAC1 variants in oocytes, SLAC1^F450A^ and SLAC1^T513D^, which mediate anion flux independently of OST1 phosphorylation (Chen et al. 2010; Maierhofer et al. 2014; Deng et al. 2021). ALMT12 inhibited both SLAC1^F450A^ (Fig. 4, D to F) and SLAC1^T513D^ (Fig. 4, G to I) activity in the absence of OST1, indicating that ALMT12 inhibits SLAC1 channel activity independently of OST1.

Collectively, these results indicate that ALMT12 interacts with and inhibits the anion channel activity of SLAC1 independently of OST1 phosphorylation.

### ALMT12 inhibits S-type anion current in guard cell

**Figure 5.**
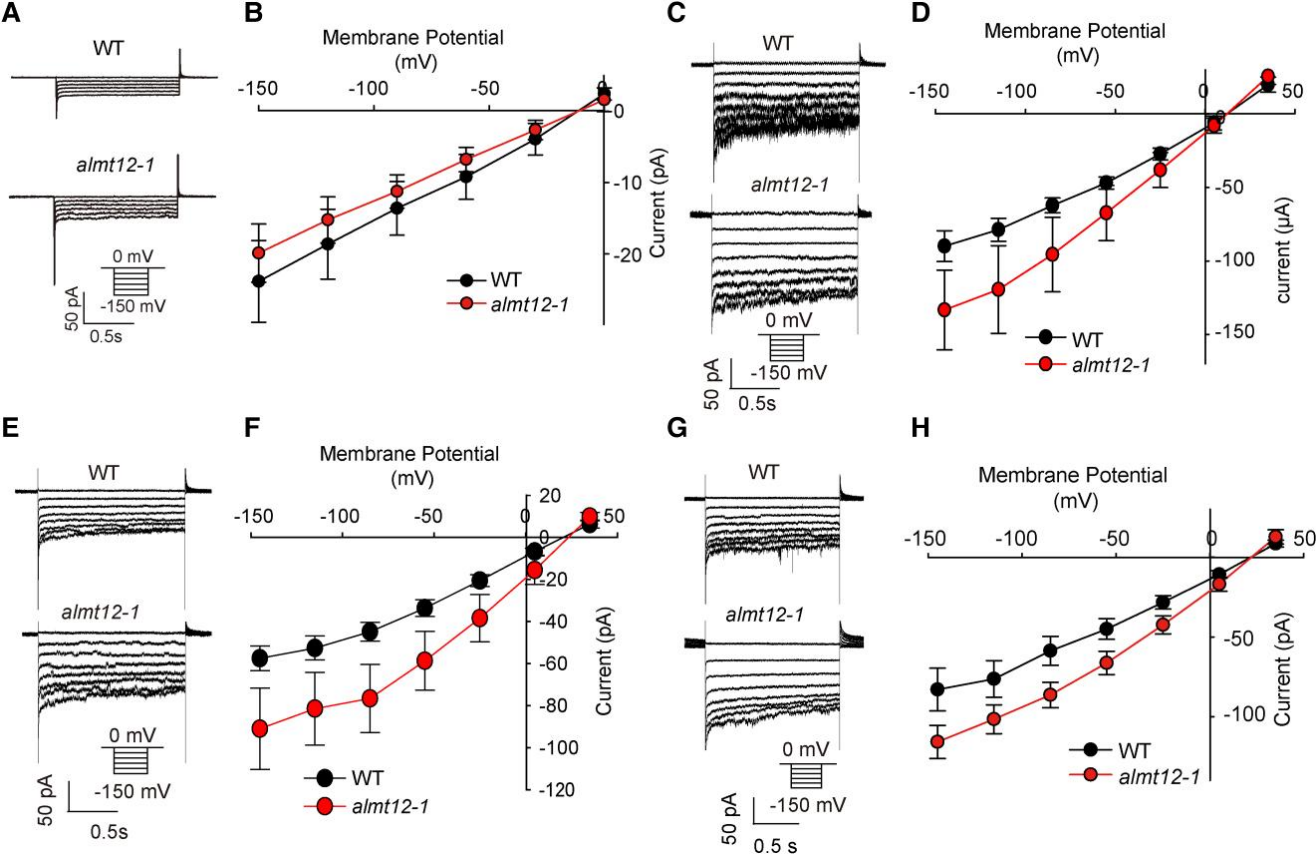
ALMT12 inhibits S-type anion current in guard cell. **A** and **B)** Patch-clamp whole-cell S-type anion channel current recordings **A)** and steady-state current-voltage relationships **B)** without treatment in WT and *almt12-1* guard cell protoplasts; The data are means ± Se (*n* = 4). **C** and **D)** Patch-clamp whole-cell S-type anion channel current **C)** and steady-state current-voltage relationships **D)** recordings with 50 *μ*M ABA added to the bath solution for WT and *almt12-1* guard cell protoplasts. The data are means ± Se (*n* = 6). **E** and **F)** Patch-clamp whole-cell S-type anion channel current **E)** and steady-state current-voltage relationships **F)** recordings with 40 mm Ca^2+^ added to the bath solution in WT and *almt12-1* guard cell protoplasts. The data are means ± Se (*n* ≥ 5). **G** and **H)** Patch-clamp whole-cell S-type anion channel current **G)** and steady-state current-voltage relationships **H)** recordings with 6.5 mm total bicarbonate added to the bath solution of WT and *almt12-1* guard cell protoplasts. The data are means ± Se (*n* ≥ 6). WT, wild-type; ABA, abscisic acid.

ALMT12 was identified as the typical R-type anion channel as evidenced by reduced R-type currents in the *almt12* mutant. Given that R-type and S-type current were previously thought to independently regulate stomatal closure. Subsequently, we employed the patch-clamp technique to directly assess the potential effects of ALMT12 on SLAC1 activity in guard cell protoplasts by recording S-type current in *almt12-1* mutant (Meyer et al. 2010) and wild-type (WT). Basal S-type anion currents were minimal in both guard cell protoplasts of WT and *almt12-1* mutant without treatment (Fig. 5, A and B). However, after 30 min ABA treatment, *almt12-1* exhibited significantly enhanced S-type anion current than WT (Fig. 5, C and D). The effects were also observed in Ca^2+^ (Fig. 5, E and F) and bicarbonate treatments (Fig. 5, G and H).

ABA, Ca^2+^, and bicarbonate induced S-type anion currents in guard cells, while ALMT12 inhibited these currents. To investigate whether stomatal closure-inducing signals (ABA, Ca^2+^, bicarbonate, or darkness) modulate the ALMT12-SLAC1 interaction, we performed LCI assays in *N. benthamiana* leaves. Notably, none of these treatments significantly altered the interaction strength between ALMT12 and SLAC1 (Supplementary Fig. S3).

The guard cell protoplast patch-clamp and interaction assays collectively demonstrate that ALMT12 inhibits SLAC1 activity under diverse conditions in guard cells.

### ALMT12 affects stomatal closure and enhances plant biomass in Arabidopsis

**Figure 6.**
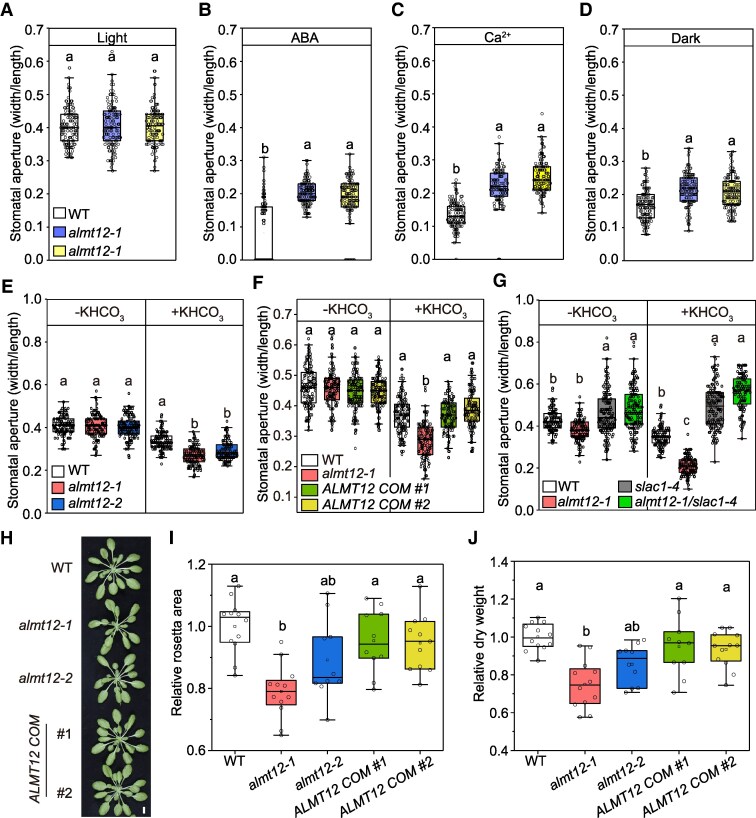
ALMT12 affects stomatal closure and enhances plant biomass in Arabidopsis. **A** to **E)** Stomatal aperture of WT, *almt12-1*, and *almt12-2* in responses to different treatments. Epidermal strips were incubated in opening buffer for 2 h in the light **A)** and then treated with 20 *μ*M ABA **B)**, 5 mm Ca^2+^  **C)**, darkness **D)**, or 10 mm KHCO_3_  **(E)** for 2 h; *n* > 120 for each material and different treatment. **F** and **G)** Stomatal aperture in WT, *almt12-1*, *ALMT12 COM*  **F)**, and *almt12-1/slac1-4*  **G)** in response to CO_2_ (10 mm KHCO_3_) for 2 h; *n* > 120 for each material and different treatment. **H** to **J)** Representative images **H)**, relative rosette area **I)**, and relative dry weight **J)** of WT, *almt12-1*, *almt12-2*, and *ALMT12 COM* plants grown under light/dark cycles under short-day conditions (150 *μ*mol m^−2^ s^−1^, 8 h light/16 h dark at 22 °C, 65% relative humidity) for 4 wks. Bar = 1 cm; *n* ≥ 10. All data are plotted with box and whiskers plots: whiskers plot represents minimum and maximum values, and box plot represents second quartile, median, and third quartile; different letters indicate significant differences at *P* < 0.05 by ANOVA with Tukey's comparison test. WT, wild-type; ABA, abscisic acid.

Inhibition of SLAC1 impairs stomatal closure, while ALMT12 plays positive role in stomatal closure as R-type anion channel. We investigated the effect of ALMT12 in stomatal closure by comparing the stomatal behavior in the 2 *almt12* mutants and WT plant in response to different signals. Initially, we observed the stomatal aperture under light condition using epidermal stripes (Fig. 6A). Then, after ABA, Ca^2+^, and darkness treatment, both mutants showed less stomatal closure than WT (Fig. 6, B to D). These data validate the role of R-type anion channel and support the previous studies (Jalakas et al. 2021). However, after 2 h bicarbonate treatment, stomata of *almt12* mutants closed more than those in WT (Fig. 6E), oppositely to other treatments. This indicates that ALMT12 might inhibit SLAC1 during CO_2_-induced stomatal closure.

To validate the function of ALMT12 in CO_2_-induced stomatal closure, we generated 2 independent complementary lines by expressing *ALMT12* driven with its own promoter in *almt12-1* mutant (*ALMT12 COM*; Supplementary Fig. S4, A and B). Using excised epidermal peel assays, we assessed the effect of bicarbonate treatment on stomatal response. Stomata from the complemented lines recovered the CO_2_ sensitivity in *almt12-1* to that level in WT plants (Fig. 6F).

Next, we generated the *almt12/slac1* double mutant (Supplementary Fig. S4, C to E) and examined its stomatal CO_2_ sensitivity alongside the single mutants with excised epidermal peels. After 2 h KHCO_3_ treatment, *almt12-1* exhibited enhanced stomatal closure than others, while both *slac1-4* and *almt12/slac1* exhibited similarly reduced stomatal closure (Fig. 6G), indicating that *SLAC1* is genetically epistatic to *ALMT12* in CO_2_-induced stomatal closure.

According to the above results, ALMT12 serves dual functions as both an R-type channel and a SLAC1 inhibitor. We propose that its role as an R-type channel promotes stomatal closure in response to darkness or drought, while its inhibitory function on SLAC1 might facilitate carbon fixation. We thus hypothesize that ALMT12 confers a growth advantage to plants by increasing CO_2_ assimilation. Indeed, the *almt12* mutants exhibited biomass reduction compared to WT, while the complementary lines restored normal growth (Fig. 6, H to J).

## Discussion

In this study, we provide multiple lines of genetic, biochemical, and electrophysiological evidence demonstrating that the guard cell R-type anion channel ALMT12 interacts with and inhibits the S-type anion channel SLAC1. This interaction and inhibition modulate stomatal movement and affect plant biomass accumulation. ALMT12 and SLAC1 are 2 types of anion channels mediating anion efflux to promote stomatal closure (Kim et al. 2010; Hedrich 2012). Although both are activated by the ABA-induced protein kinase OST1, they were previously considered as 2 independent anion efflux mechanisms. Our study uncovers a channel–channel regulation mode, similar to those reported in guard cells and roots (Zhang et al. 2016; Liu et al. 2023). In addition, SLAC1 is the core anion channel for stomatal closure in response to different signals especially in CO_2_ signal (Jalakas et al. 2021). Here, we show that ALMT12 function as a SLAC1 inhibitor to impair the stomatal closure. Additionally, the channel–channel interaction might be an extensive for channel activity regulation.

As a guard cell anion channel, ALMT12 contributes less to stomatal closure than SLAC1 (Jalakas et al. 2021). Here we propose that it might be a smart and fine-tuned regulator not just as an anion efflux mediator. Under stress conditions, ALMT12 is activated by OST1 and other kinases to promote malate efflux, fulfilling its role as an anion channel. By contrast, under elevated CO_2_ conditions, it gently inhibits SLAC1, thereby acting as a modulator of stomatal closure and enhancing plant biomass. These dual roles as a fine-tuned regulator allow balance stress responses and carbon assimilation, ultimately promoting plant biomass accumulation. However, it is still unclear the switch between the R-type channel and S-type inhibitor roles and how ALMT12 discerns CO_2_ condition. SLAC1 was reported to bind and activated by bicarbonate, whereas ALMT12 was not. Our future work will focus on 2 aspects: the CO_2_ responsiveness of ALMT12 channel activity and guard cell R-type anion current and the effect of CO_2_ on ALMT12-SLAC1 interactions in guard cells. These investigations may make elucidate the “sense” mechanism to CO_2_ concentration of ALMT12-SLAC1 complex. We propose this may be a profitable route to uncover further regulatory mechanisms of ALMT12 (similar to SLAC1); so, it is a topic that warrants further investigation outside of the scope of this study. It will expand the understanding for the activation and the CO_2_ response of these 2 anion channels.

In summary, our findings do not contradict previous reports; instead, we unveil a hidden complexity in stomatal regulation, despite this being one of the most well-studied and defined signaling networks in plant sciences. Although ALMT12 and SLAC1 are well-established components of stomatal closure, this study highlights the need to redefine the role of ALMT12 and, more broadly, anion channel regulation. We have shown that ALMT12 has a more significant role in stomatal aperture control than previously thought through its direct modulation of SLAC1 activity. We acknowledge that *almt12* mutants have a much subtler phenotype stomatal closure phenotype compared with *slac1* mutants (Jalakas et al. 2021). We propose that, this is partly due to the relief of ALMT12-mediated inhibition of SLAC1 in *almt12* mutants. However, when additional bicarbonate was applied, the *almt12* mutants performed enhanced stomatal closure, indicating that ALMT12 smartly regulates stomatal behavior: promoting closure under stress or darkness, having no effect under normal conditions, and reducing closure under elevated CO_2_. These regulatory patterns imply some unknown “sense” mechanism. Although in growth chamber, the plants face varied conditions, including variations in photoperiod and humidity. Particularly, both the atmospheric and leaf intercellular CO_2_ level dramatically change during the day in the chamber. The biomass increase might be comprehensive effects of them all. We also propose that ALMT12 modulates stomatal aperture under high CO_2_ conditions to enhance the biomass, with potential for improving CO_2_ assimilation in well-watered conditions in other species, including crop plants.

## Materials and methods

### Plant materials and growth conditions

All experiments performed on Arabidopsis (*Arabidopsis thaliana*) were in the Columbia-0 (Col-0) accession. The mutants used in this study were the T-DNA insertion mutants *almt12-1* (SM_3_38592), *almt12-2* (SM_3_1713) and *slac1-4* (SALK_137265) identified as described previously (Vahisalu et al. 2008; Meyer et al. 2010). The double mutant *slac1-4/almt12-1* was obtained by crossing the 2 single mutants. The primers for PCR genotyping of these mutants are listed in Supplementary Table S1.

For stable transformation, the vector pCAMBIA1300 was digested with the restriction enzymes NcoⅠ and PstⅠ. The full-length *ALMT12* coding sequence and the *ALMT12* promoter were amplified separately by PCR with the primer sets *ALMT12-F*/*ALMT12-R* and *ALMT12pro-F*/*ALMT12pro-R*; this was followed by overlap PCR to fuse the *ALMT12* and promoter fragment with the *ALMT12pro-F* and *ALMT12-R* primers (Supplementary Table S1). The final *ALMT12pro:ALMT12* was generated by Gibson assembly and then introduced into Arabidopsis *almt12-1* using *Agrobacterium* (*Agrobacterium tumefaciens*) strain GV3101 to generate complementation *ALMT12 COM* lines. Seeds were surface sterilized, germinated, and grown on half-strength Murashige and Skoog (MS; M519, MS basal medium with vitamins) medium with 0.8% (w/v) agar for 7 d and transferred to soil substrate (soil: vermiculite = 2:1, w/w) for growth under long-day conditions (150 *μ*mol m^−2^ s^−1^, 16 h light/8 h dark) at 22 °C. Four to 5-week-old plants grown in short-day conditions (150 *μ*mol m^−2^ s^−1^, 8 h light/16 h dark, 22 °C) were used for stomatal movement assays. *N. benthamiana* plants were grown on soil substrate in long-day conditions (400 *μ*mol m^−2^ s^−1^, 16 h light/8 h dark, 25 °C).

### Bicarbonates treatments

Bicarbonates were involved for CO_2_/bicarbonates comprehensive treatment in oocytes, guard cell protoplasts, and epidermal peels. The CO_2_ concentration could be calculated with Henderson–Hasselbalch equation: pH = pK1 + log [HCO_3_^−^]/[CO_2_] (Wang et al. 2016; Speight 2017).

### Two-electrode voltage-clamp in *Xenopus oocytes*

The full-length coding sequences of *OST1* and *SLAC1* were individually cloned into the oocyte BiFC-expression vectors pGXBG-YFP^N^ and pGXBG-YFP^C^, respectively. The coding sequences of *ALMT12*, *SLAC1^F450A^*, *SLAC1^T513D^*, *ALMT12^N^*, *ALMT12^C^*, and *ALMT1* were cloned into the vector pGXBG. The final constructs were generated by Gibson assembly and all primers are listed in Supplementary Table S1.

*X. laevis* oocytes were digested with collagenase A (5 mg/mL) (Roche Diagnostics, catalog no. 11088793001) to remove the follicular membrane. cRNAs were transcribed in vitro with a T7 Ribo MAXTM Large Scale RNA Production System (Promega). Then, total of 23 ng cRNA, with same amount of SLAC1 (8.33 ng) for each group, was injected into each oocyte and incubated in ND96 buffer at 18 °C for 2 d prior to TEVC recordings. The ND96 buffer contained 10 mm MES/Tris pH 7.5, 1 mm CaCl_2_, 1 mm MgCl_2_, 2.5 mm sodium pyruvate, 100 *μ*g/mL gentamicin sulfate, 50 *μ*g/mL streptomycin, and 96 mm NaCl (Maksaev and Haswell 2015). Whole-cell ionic currents were recorded with an Axoclamp 900A computer-controlled microelectrode amplifier and digitized using an Axon Instruments Digidata 1550 low-noise data acquisition system (Molecular Devices) controlled by pClamp acquisition software (Molecular Devices). TEVC recordings were performed with 2,000× gain, 20-kHz acquisition rate, and 500-Hz low-pass 4-pole Bessel filtering applied. The bath solution consisted of 96 mm NaCl, 1 mm CaCl_2_, 1 mm MgCl_2_, 1 mm LaCl_3_, and 10 mm MES/Tris pH 5.8 (Zhang et al. 2018). The holding potential was clamped to −20 mV and currents were elicited using a voltage step protocol ranging from +60 mV to −120 mV or −160 mV, with −20 mV step.

### Patch clamp of guard cell protoplasts

Arabidopsis guard cell protoplasts were prepared from rosette leaves of 4 to 6-wk-old plants grown in short days by enzymatic digestion. Six to 8 detached leaves were blended 3 times with deionized water at room temperature for approximately 30 s, and epidermal tissue was collected using a 74-μm nylon mesh. Mesophyll cells were separated for 1 h at 25 °C on a shaker set to 100 rpm by digestion in 1 mL enzyme solution, containing 0.7% (w/v) cellulysin (EMD Millipore Corp.), 0.1% (w/v) PVP-40, 0.25% (w/v) BSA, 0.275 mm CaCl_2_, 0.275 mm MgCl_2_, 5.5 *μ*M KH_2_PO_4_, 0.275 mm ascorbic acid, and 303 mm sorbitol, with the pH adjusted to 5.5 with Tris. After filtration through a 48-μm nylon mesh, epidermal tissue was collected and digested a second time in 1 mL enzyme solution containing 0.0075% (w/v) PET-Y23 (YAKULT HONSHA CO), 1.3% (w/v) RS cellulase (YAKULT PHARMACEUTICAL IND. CO), 0.25% (w/v) BSA, 0.5 mm CaCl_2_, 0.5 mm MgCl_2_, 10 *μ*M KH_2_PO_4_, 0.5 mm ascorbic acid, and 550 mm sorbitol, with the pH adjusted to 5.5 with Tris to release protoplasts after a 1.5 h digestion at 23 °C on a shaker set to 50 rpm. After filtration through an 18-μm nylon mesh, guard cell protoplasts were collected by centrifugation at 800 rpm for 5 min at 4 °C. The protoplasts were washed twice with wash solution (0.5 mm CaCl_2_, 0.5 mm MgCl_2_, 10 *μ*M KH_2_PO_4_, 0.5 mm ascorbic acid, and 550 mm sorbitol, with the pH adjusted to 5.5 with Tris). Guard cell protoplasts were resuspended in wash solution and incubated on ice prior to patch clamping. Currents were collected using a Multiclamp 700B amplifier (Axon instruments) and digitized using an Axon Instruments Digidata 1550 low-noise data acquisition system (Molecular Devices) controlled by pClamp acquisition software (MolecularDevices); the patch-clamp recordings were performed with 200 × gain, 20-kHz acquisition rate, and 2-kHz low-pass 4-pole Bessel filtering applied.

The pipette solution contained 150 mm CsCl, 2 mm MgCl_2_, 6.7 mm EGTA, 5 mm Mg-ATP, 10 mm MES/Tris, pH 7.1, and 5.86 mm CaCl_2_ to result in 2 *μ*M free Ca^2+^. The bath solution contained 30 mm CsCl, 2 mm MgCl_2_, 1 mm CaCl_2_, and 10 mm MES/Tris, pH 5.6 (Maksaev and Haswell 2015). To analyze S-type channels activated by bicarbonate, the guard cells were incubated in 6 mm CsHCO_3_-containing solution for 30 min before patch clamping (Hu et al. 2010). For the Ca^2+^-mediated activation of S-type anion channels, guard cells were preincubated in a bath solution with 40 mm CaCl_2_ added prior to patch clamping (Allen et al. 2002). For ABA-elicited activation of S-type channels, 50 *μ*M ABA was added to the bath solution prior to patch clamping (Mori et al. 2006). Osmolarities of the pipette and bath solutions were adjusted with sorbitol to 500 and 484 mOsmol kg^−1^, respectively. The membrane voltage was stepped from the holding potential of 0 mV to −150 mV in −30 mV decrements. All the recordings were performed with more than 5 GΩ seal.

### Y2H assay

The coding sequence encoding the C-terminal region of *ALMT12* (bp 631 to 1,683) was cloned into the vector pGADT7, while the sequence encoding the N-terminal region of *SLAC1* (bp 1 to 564) was cloned into the vector pGBKT7. Positive yeast transformants were selected on synthetic defined (SD)/-Leu-Trp medium and then re-streaked on selective medium (SD/-Leu-Trp-Ade-His). To determine the interaction intensity, 10-fold serial dilutions of saturated yeast cultures (10^0^, 10^−1^, and 10^−2^) were spotted onto selective medium and grown at 28 °C for 3 d. Primers used for the cloning of Y2H constructs are listed in Supplementary Table S1.

For membrane-based split-ubiquitin Y2H system, the coding sequence of *ALMT1*, *SLAC1* (or *SLAC1^TM^*), and *ALMT12* (or *ALMT12^N^*, *ALMT12^C^*) fused to C-terminal (Cub) or N-terminal (NubG, replacement of Ile-13 of WT NubI by glycine) of ubiquitin. After transforming the different plasmid combines to AP4 yeast, and the next steps were the same with Y2H assay. Primers used for the cloning of Y2H constructs are listed in Supplementary Table S1.

### BiFC and FLIM-FRET assays

The coding sequences of *SLAC1* and *ALMT12* were cloned into the vector pSPYNE or pSPYCE. As negative controls, the coding sequence of *ALMT1* was cloned into pSPYCE and the coding sequence of the positive control *OST1* into pSPYNE. All constructs were transformed into *Agrobacterium* strain GV3101. Combinations of agrobacteria harboring *SLAC1-YN* and *ALMT12-YC*, *ALMT12-YN* and *OST1-YC*, *SLAC1-YN* and *ALMT1-YC*, or *OST1-YN* and *SLAC1-YC* were transiently infiltrated into *N. benthamiana* leaves. The plants were cultured for 48 h, and the fluorescence images were captured using a Zeiss LSM 980+ Airyscan confocal microscope (Heidenheim, Germany). Primers used for the BiFC assays are listed in Supplementary Table S1.

For FLIM-FRET assay, SLAC1 was fused with GFP, and ALMT12 was fused with DsRed. The recombinant plasmids were transiently expressed independently or co-expressed into *N. benthamiana* leaves. The plants were cultured for 48 h, and the fluorescence images were captured using a Nikon A1 MP (Nikon, Japan), and data were collected and analyzed using the SymphoTime 64 software (PicoQuant).

### Co-IP assay

The *SLAC1* coding sequence was cloned into pCAMBIA1300 carrying the *GFP* sequence to generate *SLAC1-GFP*; the *ALMT12* coding sequence was cloned into pCAMBIA1307 harboring a sequence encoding the MYC tag to obtain *MYC-ALMT12*. All constructs were transformed into *Agrobacterium* strain GV3101 and then co-infiltrated into *N. benthamiana* leaves in the appropriate combinations. Total proteins were extracted using RIPA buffer (50 mm Tris-HCl, pH 7.4, 150 mm NaCl, 1% [v/v] NP-40, 0.25% [v/v] sodium deoxycholate, 1 mm phenylmethylsulfonyl fluoride [PMSF] and 1× protease inhibitor cocktail) and centrifuged at 12, 000 rpm for 15 min at 4 °C. The supernatant was incubated with 30 *μ*L anti-MYC antibody-magnetic agarose beads (MBL, M047-10) for 3 h at 4 °C. Bound proteins were washed 3 times with extraction buffer at 4 °C and detected by immunoblotting with anti-MYC (Abclonal, AE010) and anti-GFP antibodies (Abclonal, AE012). Primers used for the Co-IP assays are listed in Supplementary Table S1.

### LCI assay

The coding sequences of *ALMT12* and *SLAC1* were cloned in-frame with those of *LUC^N^* and *LUC^C^*, respectively. The plasmids were then transformed into *Agrobacterium* strain GV3101 for transient infiltration of *N. benthamiana* leaves. After 2 d of culture in the light, LUC activity was determined on a Tanon (5,200 multi) imaging system. Primers used for the LCI assays are listed in Supplementary Table S1.

### Stomatal aperture measurements

Stomatal apertures were measured on epidermal peels excised from the abaxial side of leaves from 3- to 4-wk-old plants. To detect CO_2_-, ABA-, CaCl_2_-, and dark-induced stomatal closure, epidermal peels were pre-incubated in stomatal opening buffer (50 mm KCl, 0.1 mm CaCl_2_, and 10 mm MES/Tris, pH 6.15) in the light (150 *µ*mol m^−2^ s^−1^) for 2 h. Then 10 mm KHCO_3_, 20 *μ*M ABA or 5 mm CaCl_2_ was freshly added to the opening buffer, and the stomata were imaged using an inverted microscope (CX43; Olympus, Tokyo, Japan) at 40× magnification. The stomatal aperture was analyzed by the width/length index of stomatal pore.

### Reverse transcription quantitative PCR analysis

Total RNA was extracted from epidermal peels rich in stomata using TRIzol reagent (Invitrogen), and first-strand cDNAs were synthesized with a SuperScript II RNase H2 reverse transcriptase kit (Invitrogen). Quantitative PCR (qPCR) was carried out using Power SYBR Green PCR Master Mix (4368577; Applied Biosystems) on a LightCycler 480 instrument (Roche) following the manufacturer's protocol. The expression of each gene was normalized to that of *ACTIN2*. qPCR was performed in triplicates; primers used for the qPCR assays are listed in Supplementary Table S1.

### Protein extraction and western blot analysis

To detect protein abundance and stability, total proteins were extracted in RIPA buffer (50 mm Tris-HCl, pH 7.4, 150 mm NaCl, 1% [v/v] NP-40, 0.25% [v/v] sodium deoxycholate, 1 mm PMSF, and 1 × protease inhibitor cocktail), mixed well, and centrifuged at 12, 000 rpm for 15 min at 4 °C. Total protein concentration was determined using a Bradford assay. The same amount of protein for each sample was separated by SDS-PAGE. Immunoblotting was carried out using anti-GFP or anti-MYC antibodies. Rubisco was used as loading control.

The western blot for oocytes product protein was followed with previous reported (Zhang et al. 2016), the coding sequence of *SLAC1* was fused upstream of 3 × *Flag* in the pGXBG-YFP^c^ vector, and the coding sequence of *ALMT1*, *ALMT12*, *ALMT12^N^*, and *ALMT12^C^* was fused upstream of 5 × *Myc* in the pGXBG vector. The immunoblot analysis followed the same method as previous reported (Zhang et al. 2016).

### Subcellular localization assay

The constructs (*SLAC1-GFP*, *MYC-ALMT12*, and *MYC-ALMT1*) were introduced into *Agrobacterium* strain AGL0 and then individually infiltrated or co-infiltrated in *N. benthamiana* leaves. A construct encoding the N-terminal of *CBL1* fused with *DsRed* (*CBL1n-DsRED*) was expressed in *N. benthamiana* leaves as a marker for membrane localization (Batistic et al. 2008). Two days after infiltration, fluorescence images were captured using a Zeiss LSM 980+ Airyscan confocal microscope (Heidenheim, Germany). Primers used are listed in Supplementary Table S1.

### Quantification and statistical analysis

No statistical methods were used to predetermine sample size. Data for quantification are presented as means ±Se or as box-and-whisker plots: whiskers represent minimum and maximum values, and boxes represent the second quartile, median, and third quartile. Statistical analyses were performed by 1-way ANOVA. The number of biologically independent replicates is indicated in the figure legends.

### Accession numbers

Sequence data from this article can be found in the GenBank/EMBL data libraries under accession numbers: *ALMT12* (AT4G17970), *SLAC1* (AT1G12480), *OST1* (AT4G33950), *ALMT1* (AT1G08430), *KAT1* (AT5G46240), and *CBL1* (AT4G17615).

## Supplementary Material

kiaf460_Supplementary_Data

## Data Availability

All data needed to evaluate the conclusions in the paper are present in the paper and/or the supplementary materials. Arabidopsis mutants used in this study are available from the corresponding authors upon request.

## Supplementary data

The following materials are available in the online version of this article.

**Supplementary Figure S1.** Both transmembrane and cytoplasmic domains are important for ALMT12-mediated SLAC1 inhibition.

**Supplementary Figure S2.** ALMT12 does not affect SLAC1 protein abundance or subcellular localization.

**Supplementary Figure S3.** ALMT12 interacts with SLAC1 independently of external conditions.

**Supplementary Figure S4.** Identification of single and double mutants by PCR and reverse transcription quantitative PCR.

**Supplementary Table S1.** List of the primers used in this study.
