# Supplementary material for: ALMT12 interacts with and inhibits SLAC1 to modulate stomatal movements and enhance plant biomass
Source: Plant Physiol. 2025 Sep 29;199(2):kiaf460. doi: 10.1093/plphys/kiaf460 (PMC12530098; doi:10.1093/plphys/kiaf460)
Supplement: kiaf460_Supplementary_Data [file kiaf460_supplementary_data.pdf]

Supplementary data

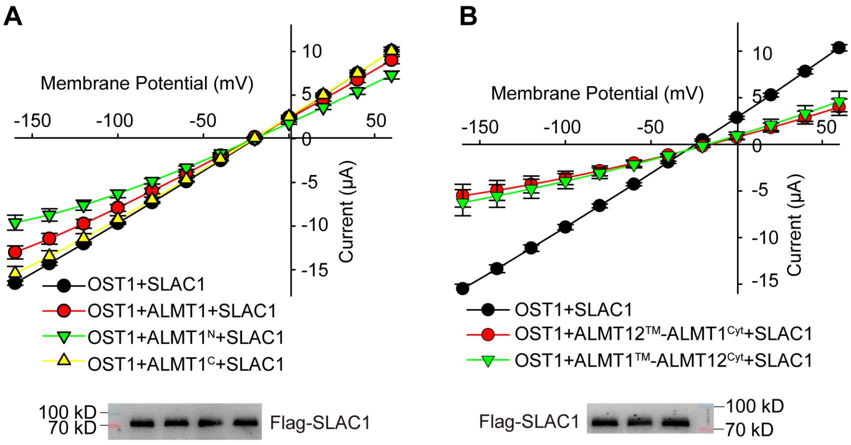

Supplementary Figure S1. Both transmembrane and cytoplasmic domains are important for ALMT12 inhibited SLAC1.

(A) Steady-state current-voltage relationships and immunoblotting analysis of oocytes co-expressing *SLAC1* and *OST1*, alone or with *ALMT1*, *ALMT1*^{N} or *ALMT1*^{C}. The data are means ± SE ($n \geq 10$).

(B) Steady-state current-voltage relationships and immunoblotting analysis of oocytes co-expressing *SLAC1* and *OST1*, alone or with *ALMT12*^{TM}*-ALMT1*^{Cyt}, or *ALMT1*^{TM}*-ALMT12*^{Cyt}. The data are means ± SE ($n \geq 10$). Cyt: Cytosolic Domain; TM: Transmembrane Domain.

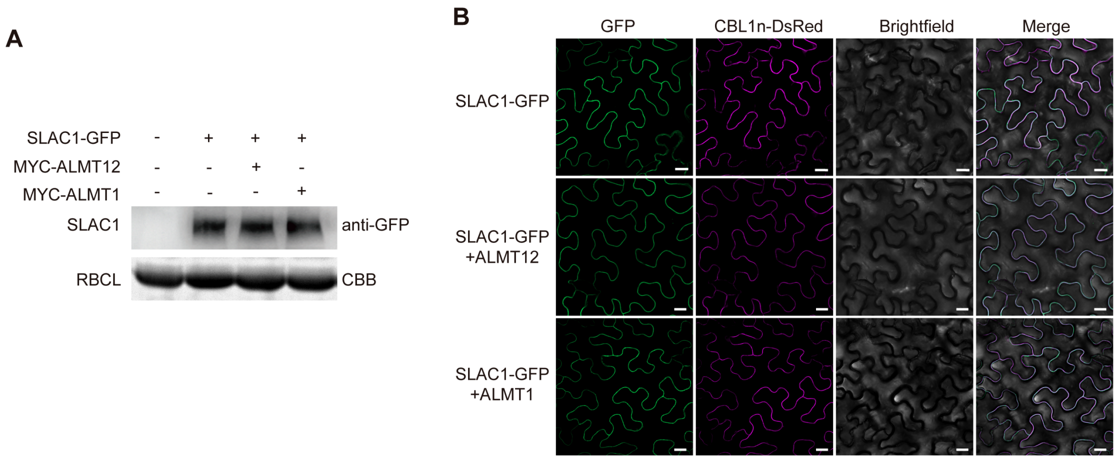

Supplementary Figure S2. ALMT12 does not affect the protein abundance or subcellular localization of SLAC1.

(A) Immunoblot analysis showing the abundance of SLAC1-GFP when *N. benthamiana* leaves were infiltrated with *SLAC1-GFP* alone or together with *ALMT12* or *ALMT1*. CBB: Coomassie Brilliant Blue.

(B) Representative images showing the subcellular localization of SLAC1-GFP alone or with ALMT12 or ALMT1 in *N. benthamiana* leaves. *CBL1n-DsRED* was co-infiltrated as a plasma membrane marker. Scale bars, 20 μm.

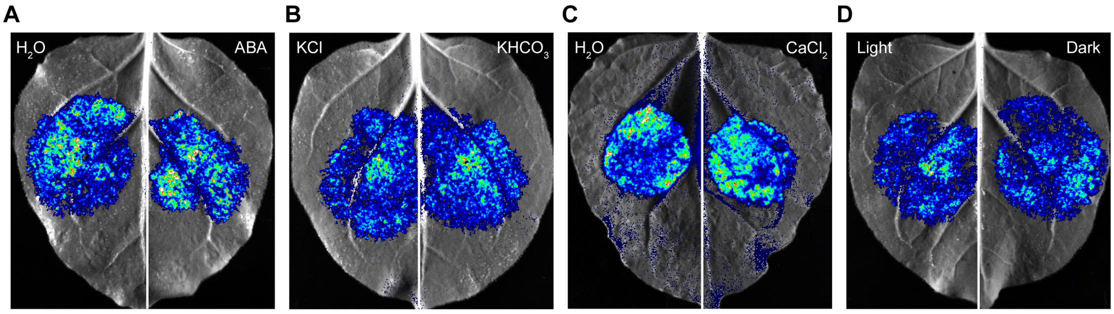

Supplementary Figure S3. ALMT12 interacts with SLAC1 independently of the external conditions. *ALMT12-LUC*^{C} and *SLAC1-LUC*^{N} were co-expressed in *N. benthamiana* leaves, and the leaves were treated with ABA **(A)**, $KHCO_3$ **(B)**, $CaCl_2$ **(C)** or dark **(D)** for 12 h before LUC luminescence detection. H_2O, KCl, and light were used as treatment controls. ABA: Abscisic Acid.

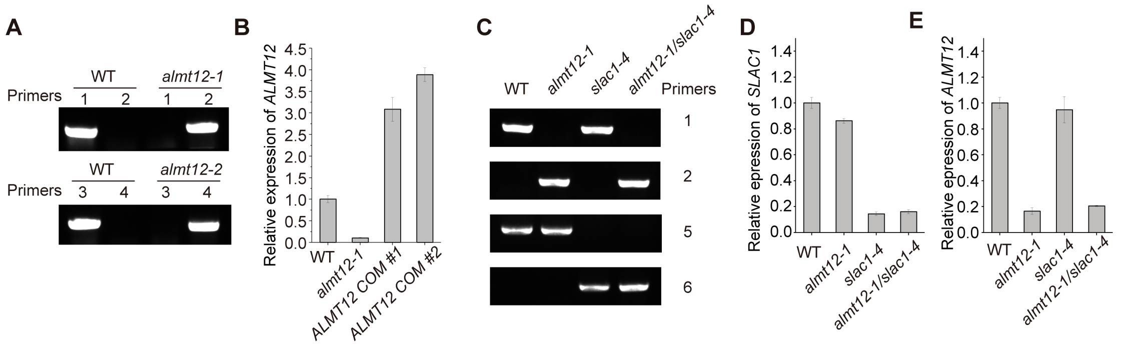

Supplementary Figure S4. Identification of single and double mutants by PCR and RT-qPCR.

(A) PCR genotyping of *almt12-1* and *almt12-2*. The PCR products were amplified using the primer pairs 1, *almt12-1-LP*+*almt12-1-RP*; 2, *almt12-1-LP*+*Spm32*; 3, *almt12-2-LP*+*almt12-2-RP*; 4, *almt12-2-LP*+*Spm32*. WT: Wild-Type.

(B) RT-qPCR analysis of *ALMT12* expression in the *ALMT12 COM* lines. Data are means ± SE (*n* = 3)

(C) PCR genotyping of the *almt12-1/slac1-4* double mutant using the primer pairs 5, *slac1-4-LP* + *slac1-4-RP*; 6, *LBB1.3* + *slac1-4-RP.*

(D and E) RT-qPCR analysis of *SLAC1* **(D)** and *ALMT12* **(E)** expression in WT, *almt12-1*, *slac1-4* single mutants, and the *almt12-1/ slac1-4* double mutant. Data are means ± SE (*n* = 3).

Supplementary Table S1 List of primers utilized in this study.

Names	Nucleotide sequence (5' → 3')
**Primers for yeast two-hybrid**	
AD-ALMT12C-F	GACGTACCAGATTACGCTATGTCTGGTGAAGATCTA
AD-ALMT12C-R	GAGCTCGATGGATCCTTATTCCGCGGCACCGACACT
BD-SLAC1N-F	ATCTCAGAGGAGGACCTGATGGAGAGGAAACAGTCA
BD-SLAC1N-R	ATGCGGCCGCTGCAGTTAGAACCTTAGGAGAAACGG
**Primers for dual membrane yeast two-hybrid**	
NubG-SLAC1-F	CCAACCACCATAGAATTCATGGAGAGGAAACAGTCA
NubG-SLAC1-F	ACAAGAAAGCTGGGTACCGTGATGCGACTCTTCCTC
Cub-ALMT12-F	ACCATGAATTCCCTGCAGATGTCCAATAAGGTTCAC
Cub-ALMT12-R	GTACTCGAGCGGAAGCTTTTCCGCGGCACCGACACT
Cub-ALMT1-F	ACCATGAATTCCCTGCAGATGGAGAAAGTGAGAGAG
Cub-ALMT1-R	GTACTCGAGCGGAAGCTTCTGAAGATGCCCATTACT
Cub-ALMT12^{C}-F	ACCATGAATTCCCTGCAGATGTCTGGTGAAGATCTA
Cub-ALMT12^{C}-R	GTACTCGAGCGGAAGCTTTTCCGCGGCACCGACACT
Cub-ALMT12^{N}-F	ACCATGAATTCCCTGCAGATGTCCAATAAGGTTCAC
Cub-ALMT12^{N}-R	GTACTCGAGCGGAAGCTTCCAAATAGGAAAAACAAG
Cub-SLAC1^{TM}-F	CACCATGAATTCCCTGCAGATGATTGGATGCTTCGGTATC
Cub-SLAC1^{TM}-R	GTACTCGAGCGGAAGCTTCCAGACAAAGGCGTGGAG
**Primers for biomolecular fluorescence complementation**	
ALMT12YC-F	AACACGGGGGACTCTAGAATGTCCAATAAGGTTCAC
ALMT12YC-R	CATCCCGGGAGCGGTACCTTCCGCGGCACCGACACT
SLAC1YN-F	AACACGGGGGACTCTAGAATGGAGAGGAAACAGTCA
SLAC1-YN-R	CATCCCGGGAGCGGTACCGTGATGCGACTCTTCCTC
**Primers for co-immunoprecipitation and subcellular localization**	
SLAC1-GFP-F	GAGAACACGGGGGACGAGCTCATGGAGAGGAAACAGTCAAATGC
SLAC1-GFP-R	CTCCTTTACTCATGTCGACGTGATGCGACTCTTCCTC
MYC-ALMT12-F	AACTCGGTATCTAGAACTAGTATGTCCAATAAGGTTCACGTAGGG
MYC-ALMT12-R	TTTGCGGAGTACCCGGGTACCTCATTCCGCGGCACCGAC
**Primers for luciferase complementation imaging**	
ALMT12-LUCC-F	GGGCGGTACCCGGGATCCATGTCCAATAAGGTTCAC
ALMT12-LUCC-R	AAAGCTCTGCAGGTCGACTTATTCCGCGGCACCGAC
SLAC1-LUCN-F	GCTCGGTACCCGGGATCCATGGAGAGGAAACAGTCA
SLAC1-LUCN-R	GTACGAGATCTGGTCGACGTGATGCGACTCTTCCTC
ALMT12-LUCN-F	GCTCGGTACCCGGGATCCATGTCCAATAAGGTTCAC
ALMT12-LUCN-R	GTACGAGATCTGGTCGACTTCCGCGGCACCGACACT
SLAC1-LUCC-F	GGGCGGTACCCGGGATCCATGGAGAGGAAACAGTCA
SLAC1-LUCC-R	AAAGCTCTGCAGGTCGACTTAGTGATGCGACTCTTC
**Primers for RT-qPCR**	
qPCR-SLAC1-F	CAGCATCCGTAGCAACAATAAAG
qPCR-SLAC1-R	GAGAAGCGTGGAGACAAACA
qPCR-ALMT12-F	TTTGACGGAACTCGCAGATAG
qPCR-ALMT12-R	ACGTGGAGATGATCGGATAGA
**Primers for two-electrode-voltage-clamp**	
XB-SLAC1-F	AAACGCTCAACTTTGGCCATGGAGAGGAAACAGTCA
XB-SLAC1-R	CAGGCTGAGGTTTAATCCGTGATGCGACTCTTCCTC
XB-ALMT12-F	AAACGCTCAACTTTGGCCATGTCCAATAAGGTTCAC

XB-ALMT12-R	GTTCTTGAGGCTGGTTTATTCCGCGGCACCGACACT
XB-OST1-F	AAACGCTCAACTTTGGCCATGGATCGACCAGCAGTG
XB-OST1-R	CAGGCTGAGGTTTAATCCCATTGCGTACACAATCTC
XB-ALMT12N-F	AAACGCTCAACTTTGGCCATGTCCAATAAGGTTCAC
XB-ALMT12N-R	GTTCTTGAGGCTGGTTTATTACCAAATAGGAAAAAC
XB-ALMT12C-F	AAACGCTCAACTTTGGCCATGTCTGGTGAAGATCTA
XB-ALMT12C-R	GTTCTTGAGGCTGGTTTATTATTCCGCGGCACCGAC
**Primers for cloning of ALMT12 and ALMT12 promoter**	
ALMT12-F	CTGAATCAAAGGCCATGGATGGTTGCAACAGAGAGG
ALMT12-R	ATACGAACGAAAGCTCTGCAGTCATTCCGCGGCACCGAC
ALMT12pro-F	CTGAATCAAAGGCCATGGGCAGTCTTGCAGACATAT
ALMT12pro-R	GTGAACCTTATTGGACATTTTGAGGGAGAGAAATTG
**Primers for genotyping**	
almt12-1-LP	GTTGTGCAAAGGGCTTAATAGAG
almt12-1-RP	CAAGAAGGCTCATGAAAAGACAG
almt12-2-LP	ACAAGACCACCGTTGGTAAACTC
almt12-2-RP	CTCCGGCTAATCTTACACAAGG
Spm32	TACGAATAAGAGCGTCCATTTTAGAGT
slac1-4-LP	TCTGCACATATGTTTGGTTGC
slac1-4-RP	TCCAATTGGGAACCTTAGGAG
LBB1.3	GCGTGGACCGCTTGCTGCAACT
